# Computational discovery of dynamic cell line specific Boolean networks from multiplex time-course data

**DOI:** 10.1371/journal.pcbi.1006538

**Published:** 2018-10-29

**Authors:** Misbah Razzaq, Loïc Paulevé, Anne Siegel, Julio Saez-Rodriguez, Jérémie Bourdon, Carito Guziolowski

**Affiliations:** 1 Université de Nantes, Centrale Nantes, CNRS, Laboratoire des Sciences du Numérique de Nantes (LS2N UMR 6004), F-44000, Nantes, France; 2 LRI UMR8623, Université Paris-Sud, CNRS, Université Paris-Saclay, F-91400 Orsay, France; 3 Université Bordeaux, Bordeaux INP, CNRS, LaBRI, UMR5800, F-33400 Talence, France; 4 Institut de Recherche en Informatique et Systèmes Aléatoires, Rennes, France; 5 RWTH-Aachen University, Faculty of Medicine, Joint Research Center for Computational Biomedicine, Aachen, Germany; 6 European Molecular Biology Laboratory, European Bioinformatics Institute (EMBL-EBI), Cambridgeshire, UK; ETH Zurich, SWITZERLAND

## Abstract

Protein signaling networks are static views of dynamic processes where proteins go through many biochemical modifications such as ubiquitination and phosphorylation to propagate signals that regulate cells and can act as feed-back systems. Understanding the precise mechanisms underlying protein interactions can elucidate how signaling and cell cycle progression occur within cells in different diseases such as cancer. Large-scale protein signaling networks contain an important number of experimentally verified protein relations but lack the capability to predict the outcomes of the system, and therefore to be trained with respect to experimental measurements. Boolean Networks (BNs) are a simple yet powerful framework to study and model the dynamics of the protein signaling networks. While many BN approaches exist to model biological systems, they focus mainly on system properties, and few exist to integrate experimental data in them. In this work, we show an application of a method conceived to integrate time series phosphoproteomic data into protein signaling networks. We use a large-scale real case study from the HPN-DREAM Breast Cancer challenge. Our efficient and parameter-free method combines logic programming and model-checking to infer a family of BNs from multiple perturbation time series data of four breast cancer cell lines given a prior protein signaling network. Because each predicted BN family is cell line specific, our method highlights commonalities and discrepancies between the four cell lines. Our models have a Root Mean Square Error (RMSE) of 0.31 with respect to the testing data, while the best performant method of this HPN-DREAM challenge had a RMSE of 0.47. To further validate our results, BNs are compared with the canonical mTOR pathway showing a comparable AUROC score (0.77) to the top performing HPN-DREAM teams. In addition, our approach can also be used as a complementary method to identify erroneous experiments. These results prove our methodology as an efficient dynamic model discovery method in multiple perturbation time course experimental data of large-scale signaling networks. The software and data are publicly available at https://github.com/misbahch6/caspo-ts.

## Author summary

Traditional canonical signaling pathways help to understand overall signaling processes inside the cell. Large scale phosphoproteomic data provide insight into alterations among different proteins under different experimental settings. Our goal is to combine the traditional signaling networks with complex phosphoproteomic time-series data in order to unravel cell specific signaling networks. In this study, we have applied the caspo time series (*caspo-ts*) approach which is a combination of logic programming and model checking, over the time series phosphoproteomic dataset of the HPN-DREAM challenge to learn cell specific BNs. The learned BNs can be used to identify the cell specific topology. Our analysis suggests that *caspo-ts* scales to real datasets, outputting networks that are not random with a lower fitness error than the models used by the 178 methods which participated in the HPN-DREAM challenge. On the biological side, we identified the cell specific and common mechanisms (logical gates) of the cell lines.

## Introduction

Protein signaling networks are static views of dynamic processes since they respond to stimuli and perturbation. They constitute complex regulatory systems controlled by crosstalk and feedback mechanism. Because these networks are often altered in diseases, discovering the precise mechanisms of signal transduction may provide a better fundamental understanding of disease behavior. For instance, a main difficulty in cancer treatment is that different signaling networks fact that cell populations specialize upon treatment and therefore patient responses may be heterogeneous. Computational models of signaling control for different patient groups could guide cancer research towards a better drug targeting system. In this work, we propose a methodological framework to discriminate among the regulatory mechanisms of four breast cancer cell lines by building predictive computational models.

Several formalisms have been used widely to model interaction networks. Models built using differential equations require explicit specifications of kinetic parameters of the system and work well for small-scale systems. While being a useful tool, mathematical modeling becomes computationally intensive as networks become larger [1–3]. Stochastic modeling is suitable for problems of a random nature but also fails to scale well with large scale systems [1].

The Boolean Network (BN) formalism [4] is a powerful approach to model signaling and regulatory networks [5]. Various BN learning frameworks exist focusing on varying levels of details [1, 6]. As compared to the extensive literature on Boolean frameworks, BN modeling of signaling networks is quite recent.

In this work, we have used the caspo time series (*caspo-ts*) [7, 8] method to learn BNs from multiple perturbation phosphoproteomic time series data given a Prior Knowledge Network (PKN). We have improved and adapted *caspo-ts* to deal with a midscale Prior Knowledge Network (PKN) with 64 nodes and 178 edges in order to learn the BNs of four breast cancer cell lines (BT20, BT549, MCF7, UACC812) from their time series phosphoproteomic datasets. Importantly, the PKN did not contain any information about the temporal changes or dynamic properties of the proteins. This information was learned from a dataset describing the dynamics of signaling processes for those breast cancer cell lines as part of the HPN-DREAM challenge. In comparison to the current methods that learn signaling networks as Boolean models using static measurements[9, 10], and one-time point measurements across multiple perturbations [11–14], our method allows us to handle time series data. A further advantage is the guarantee of discovering optimal BNs, where the distance between original and over-approximated time series is minimal. This is achieved by using computational solvers such as Answer Set Programming (ASP) [15].

Our results show that the ASP component of our method allows us to filter the explosion of possible dynamical states inherent to this type of problem, and thanks to that filtering, the model-checking step allows us to provide BNs exactly reproducing the binarized time series data. These BNs are referred to as true positive (TP) BNs. Our results point to measurements in the time series HPN-DREAM dataset that contradict the experimental setting and to perturbations that show contradictory dynamics. We observed that given the same PKN, the solving time was different for each cell line dataset. For cell lines BT20, BT549, MCF7 our method found TP BNs, while for the UACC812 cell line dataset it was impossible to find a TP BN within a time-frame of 7 days. This computation time difference is due to the different structure of the solution space among cell lines. This could point to the situation where the dataset is not explainable by the prior knowledge network, which may give valuable insights to experimentalists. For example, that the number of consistent experimental perturbations is not sufficient, and that the knowledge of the PKN is incomplete given this dataset. We also show that this method is capable of recovering time series measurements with a Root Mean Square Error (RMSE) of 0.31, the minimum achieved so far as compared to other participants of the HPN-DREAM challenge. Our method focuses on learning optimal BNs’ structures. It does not predict time-series traces of the proteins from the learned BNs. However it detects the minimum distance that is possible to obtain from the proteins of the learned BNs in comparison to the time-series traces in the testing data. This is the main conceptual difference of our method compared to those proposed by the HPN-DREAM challenge. This difference needs to be considered when comparing the RMSE score. Based on a comparison with the canonical mTOR pathway, we show that the discovered context specific BNs have an average AUROC score of 0.77. We found 38% of the cell line specific interactions explaining the heterogeneity among these four cancer cell lines, which can be observed in different cell line specific networks, shown in S1, S2, S3 and S4 Figs. All in all, our results show that *caspo-ts* handles real (HPN-DREAM) datasets, where data points are incomplete and subject to experimental error. Our method is applicable to any kind (gene or protein expressions) of time series datasets measured upon different perturbations. We have proved here that *caspo-ts* handles a PKN size of 64 nodes and 170 edges; this is relevant since approaches modeling time usually only scale up to very small networks because of state graph explosion.

### Related work

Regarding the training of BNs with respect to multiple perturbation datasets, CellNOpT (CNO)[16] assembles BNs from a Prior Knowledge Network (PKN) and phosphoproteomic datasets. Their tool has been implemented using stochastic search algorithms (more precisely, a genetic algorithm), to suggest multiple BNs explaining the data [17]. However, stochastic search methods cannot generate a complete set of solutions, hence they cannot guarantee a global optimal solution. In [11, 12], the authors overcome this problem by proposing *caspo*, an approach based on ASP to infer BNs explaining the underlying protein signaling network. This approach can generate all possible optimal Boolean models as compared to the CellNOpt approach. The authors in [14], presented a framework based on integer linear programming (ILP) to learn the subset of interactions best fitted to the experimental data. Recently, another approach based on ILP has been proposed to reconstruct BNs from experimental data. Their learning approach do not require the information about the activation/repression properties of the network’s edges [13].

The methods mentioned above are very useful but restrain themselves to learn from only two time points, assuming the system has reached an early steady-state when the measurements are performed. This assumption prevents us from capturing interesting characteristics like loops [3]. To overcome this issue, the caspo time series (*caspo-ts*) method was proposed in [8]. This method learns BNs from multiple perturbation phosphoproteomic time series data given a PKN. The proposed method is based on ASP and a model-checking step is needed to detect true positive BNs. They tested their approach on synthetic data for a small PKN (≈17 nodes and ≈50 edges) [8]. More recently, an approach based on genetic algorithms was proposed to learn context specific networks given a PKN and experimental information about stable states and their transitions but it does not scale well with large networks and finding a global optimum is not guaranteed [18].

#### *Caspo-ts* modeling framework

We chose the *caspo-ts* method [7, 8] for the inference of BNs. This method was tailored to handle protein phosphoproteomic time series data. The input of the method consists of a PKN and normalized phosphoproteomic time series data under different perturbations to generate a family of BNs whose structure is compatible with the PKN and that can also reproduce the patterns observed in the experimental data. In the following, we will develop the main notions of this framework.

#### Prior knowledge network

**Fig 1:**
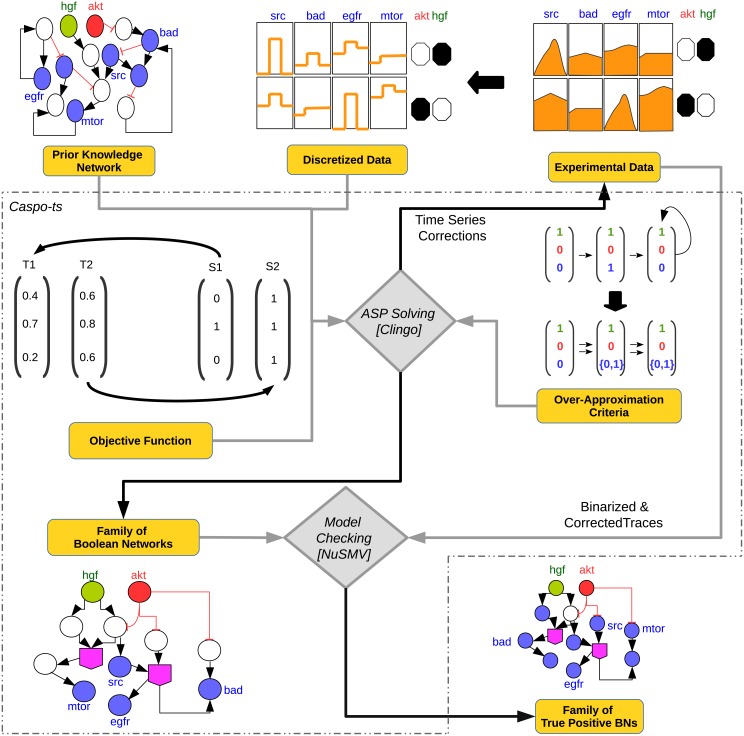
*Caspo-ts* workflow. *Caspo-ts* receives as input data a prior knowledge network (PKN) and a discretized phosphoproteomic dataset. In this example the phosphoproteomic data consists of two perturbations involving *akt* (inhibitor) and *hgf* (stimulus): 1) *akt* = 0, *hgf* = 1 and 2) *akt* = 1, *hgf* = 0. A black colored perturbation means the inhibitor or stimulus was perturbed (1) while white represents the opposite (0). Readouts are specified in blue and describe the time series under given perturbations. Using this input data, *caspo-ts*, performs two steps: ASP solving and model checking. In the ASP solving step: (i) a set of BNs, compatible with the PKN, is generated, (ii) afterwards an *over-approximation constraint* is imposed upon each candidate BN to filter out invalid BNs, that do not result in an over-approximation of the reachability between the Boolean states given by the phosphoproteomic dataset, and finally (iii) BNs are optimized using an objective function minimizing the distance to the experimental measures. The ASP step also introduces repairs in some data points of the time series that added penalties to the objective function. These corrected traces will be given to the model checker. In the model checking step, the exact reachability of all the (binarized and corrected) time series traces in the family of BNs is verified.

It is one input of *caspo-ts* and it is modeled as a labeled (or colored) directed graph (*V*, *E*, *σ*) with *V* = {*v*_1_, *v*_2_, …, *v*_*n*_} the set of nodes, *E* ⊆ *V* × *V* the set of directed edges and *σ* ⊆ *E* × {+1, −1} the signs of edges. The set of nodes is denoted by *V* = *S*∪*I*∪*R*∪*U* where *S* are stimuli, *I* are inhibitors, *R* are readouts, and *U* are unobserved nodes. Stimuli, inhibitors, readouts, and unobserved nodes are encoded by different colors in the graphs presented in this case study. Stimuli are shown in green, inhibitors in red, readouts in blue, and unobserved nodes in white (Fig 1). Moreover, the subsets *S*, *I*, *R*, *U* are all pairwise disjoint except for *I* and *R*, because a protein can be inhibited as well as measured. Stimuli are used to bound the system and also serve as interaction points of the system, these nodes can be experimentally stimulated, *e*.*g*. cellular receptors. Inhibitors are those nodes which remain inactive or blocked over all time points of the experiment by small molecule inhibitors. Stimuli and inhibitor nodes take Boolean values {0, 1} representing the fact that the node was stimulated (1) or inhibited (0). Readouts are experimentally measured given a combination of stimuli and inhibitors. They usually take continuous values in [0;1] after normalization. Unobserved nodes are neither measured nor experimentally manipulated. In this study, we use the term *perturbation* to refer to the combination of stimuli and inhibitors, similarly to other studies such as [19–21].

#### Phosphoproteomic time series data

It is the second input of *caspo-ts* and it consists of temporal changes in phosphorylated proteins under a perturbation (Fig 1). Without loss of generality, we assume that the time series data are related to the observation of *m* ≤ *n* nodes for the nodes {*v*_1_, …, *v*_*m*_} (so the nodes {*v*_*m*+1_, …, *v*_*n*_} are not observed). The observations consist of normalized continuous values: a time series of k data points is denoted by $T_{P}=\left(t_{P}^{1},\ldots ,t_{P}^{k}\right)$, where *P* ⊆ *S* ∪ *I* is a perturbation and *t*^*j*^ ∈ [0; 1]^*m*^ for 1 ≤ *j* ≤ *k*. This data will be discretized in order to link it with further BNs’ discovery (ASP solving and model checking steps).

#### Boolean Network

It is the output of *caspo-ts*. A *Boolean Network (BN)* [22, 23] is defined as a pair *B* = (*N*, *F*), where

- *N* = {*v*_1_, …, *v*_*n*_} is a finite set of nodes (or variables/proteins/genes)
- *F* = {*f*_1_, …, *f*_*n*_} is a set of Boolean functions (regulatory functions) $f_{i}:B^{k}\to B$, with B={0,1}, describing the evolution of variable *v*_*i*_.

A vector (or *state*) *x* = (*x*_1_, …, *x*_*n*_) captures the values of all nodes *N* at a time step, where *x*_*i*_ represents the value of the node *v*_*i*_, and is either 1 or 0. There are up to 2^*n*^ possible distinct states for each time step. Next, we define the transition *x* → *x*′ between two states of a BN. If there is no update for node *v*_*i*_ then $x_{i}^{\prime }$ = *x*_*i*_. If there is an update for node *v*_*i*_ then the state of a node *v*_*i*_ at the next time step is determined by $x_{i}^{\prime }=f_{i}\left(x_{1},\ldots ,x_{n}\right)$. Note that usually only a subset of the nodes influence the evolution of node *v*_*i*_. These nodes are called the *regulatory nodes* of *v*_*i*_. The state of each node can be updated in a synchronous (parallel) or asynchronous fashion. In the synchronous update schedule, the states of all nodes are updated, while in asynchronous update schedule, the state of one node is updated at a time. The work presented in this article is independent of the update schedule routine, hence any number of nodes can be updated at a time.

#### ASP solving

Given a PKN and a phosphoproteomic dataset, a family of candidate BNs, compatible with this PKN, is exhaustively enumerated including the main nodes (the sets *S*,*I*,*R*) of the experimental data. We refer the reader to [12] for a detailed description of BN’s compatibility with a PKN. Afterwards an *over-approximation constraint* (see Materials and methods) is imposed upon each candidate BN to filter out invalid BNs [8], that do not result in an over-approximation of the reachability between the Boolean states given by the phosphoproteomic dataset. Finally, an optimization step is performed to select those BNs having a minimal distance between the actual time series *T*_*P*_ and the over-approximated time series *Y*_*P*_. We have adopted the Root Mean Square Error (RMSE) as the *objective function*:

$$\begin{array}{c} RMSE=\sqrt{\frac{1}{m*k*|P|}\sum_{i=1}^{m}\sum_{j=1}^{k}\sum_{P\in P}{\left({\left(t_{P}^{j}\right)}_{i}-{\left(y_{P}^{j}\right)}_{i}\right)}^{2}} \end{array}$$ (1)

where *m* is the number of observed nodes, *k* is the number of time points, and P is the set of perturbations. In addition, the optimization step highlights the data points in the time series which added penalties to the RMSE. Such data points are automatically corrected before the model checking step.

All the analyses described in this step are performed using ASP, namely the clingo 4.5.4 solver [15]. This solver guarantees finding optimal solutions, and all BNs outputted by the ASP solver step will be identically optimal. For the HPN-DREAM case study, the full enumeration of optimal BNs creates billions of BNs, and since the next (model checking) step can take days of computation depending on the verified BN we choose to limit this enumeration to a fixed number of BNs.

#### Model checking and true positive BNs

From the ASP solving step, a set of optimal BNs that over-approximate the phosphoproteomic time series data is produced. This set of BNs is verified with an exact *model checking* to detect true positive (TP) BNs. *TP BNs* are guaranteed to reproduce all the (binarized) trajectories under all perturbations by verifying exact reachability in the BN state graph. For this, we have used computational tree logic (CTL) implemented in the NuSMV 2.6.0 [24], which is a symbolic model checker.

#### Caspo-ts workflow

The *caspo-ts* workflow is shown in Fig 1. It consists of two main steps, ASP solving and model checking, as described previously.

## Results

### Prior knowledge network

**Fig 2:**
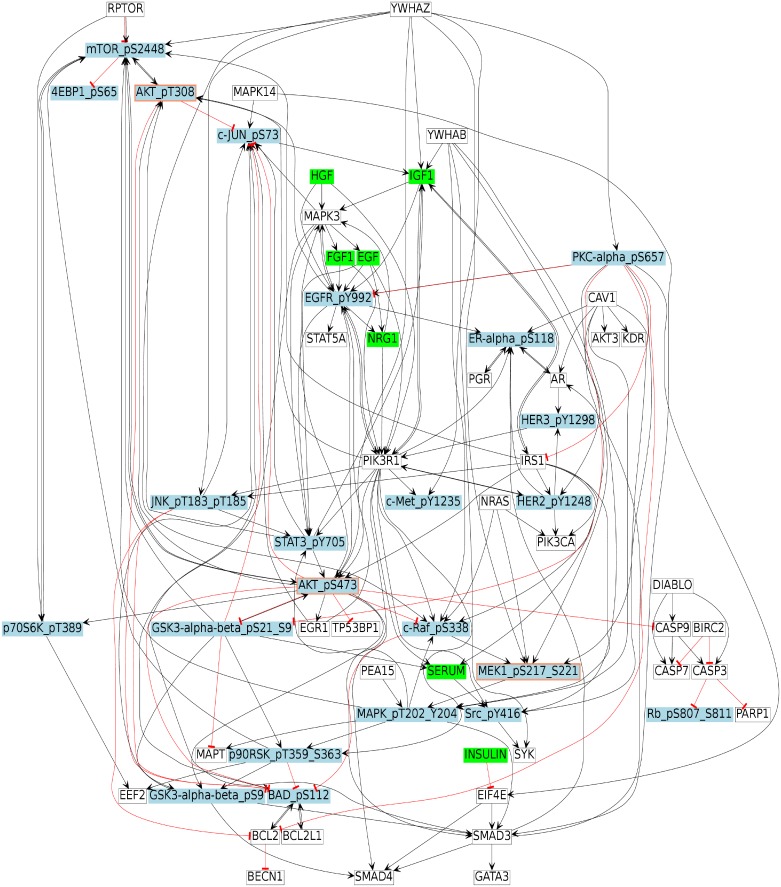
Breast cancer signaling pathway. This figure shows the reconstructed signaling network from a combination of databases. An arrow shows the positive regulatory relationship between two proteins, while a T shaped arrow indicates inhibition. Green nodes are stimuli, blue nodes are readouts, white nodes are unmeasured or unobserved, and blue nodes with a red border represent inhibitors and readouts at the same time. Please note that in the node labels, we have added the phosphorylation sites to the protein names in order to connect them to the experimental measurements.

The structure of the protein signaling network was generated by mapping the experimentally measured phosphorylated proteins (HPN-DREAM dataset) to their equivalents from literature-curated databases and connecting them together within one network (see Materials and methods). The reference network (Fig 2) was built using the ReactomeFIViz app (also called the ReactomeFIPlugIn or Reactome FI Cytoscape app) [25], which accesses the interactions existing in the Reactome and other databases [25, 26]. The PKN shown in Fig 2 consists of 64 nodes (7 stimuli, 3 inhibitors, and 23 readouts) and 178 edges.

### Data processing

The learning and testing datasets used in this study were extracted from the HPN-DREAM challenge and correspond to time series protein measurements upon different perturbations of four breast cancer cell lines—UACC812, BT20, BT549, and MCF7 [20, 21] (see Materials and methods). Since readout signals are measured on variable ranges depending on the protein, a normalization step was necessary. The learning dataset had a few noisy, inconsistent and incomplete time series data points. The *caspo-ts* system identified these inconsistencies existing in the time series data. The recurrent experimental inconsistency observed was an oscillation in the protein signal upon experimental inhibition of the same protein.

To resolve the above mentioned issues, we performed the following data processing steps on the learning dataset:

1. Set the protein values between a common scale, *i*.*e*., 0 and 1, using a maximum-value-based normalization scheme (see Materials and methods).
2. For time point 0 the expression of some readout proteins under some perturbations was not available. Thus, control experimental readings have been used as the time point 0 for such proteins.
3. In some cases readout measurements were duplicated for the same time point, to solve this noise issue we have chosen one time point arbitrarily.
4. We have removed inconsistent perturbations where the protein AKT was inhibited and was having a dynamic behavior as a readout protein.
5. We have considered only perturbations with complete time series data, since guessing the missing time points automatically with *caspo-ts* for this case study will be computationally expensive.

The experimental errors pointed in steps 2-5 were raised as warning or exceptions by *caspo-ts*. Steps 1 to 5 were applied on the learning dataset. Only step 1 was applied on the testing dataset.

### Cell line specific Boolean Networks

In this section, we show the generated BNs for each cell line. For this, we used *caspo-ts* to learn the BNs from the PKN (Fig 2) and the phosphoproteomic data of four breast cancer cell lines—BT20, BT549, MCF7, and UACC812. We inferred a family of cell line specific BNs for each cancer cell line and they are shown in the Supplementary Figures (S1, S2, S3 and S4 Figs).

As explained in the *caspo-ts modeling framework* section, the *caspo-ts* method produces BNs fulfilling two criteria, (i) satisfaction of the over-approximation criteria (see Materials and methods) and (ii) optimality with respect to the RMSE objective function. ASP-optimal solutions were fast to collect, their computation time ranged from 36 seconds to 3 minutes depending on the cell line (S1 Table). Afterwards, these ASP-optimal BNs were given to the model-checker for further verification. This second step is more complex and we put a restriction for the computation time of 7 days for each cell line. The number of verified BNs varies from one cell line to another, depending on a number of factors such as the number of perturbations, the order of answer sets in the solutions space, and the perturbation order. The total number of verified ASP-optimal BNs within the 7 days time-frame were 231, 52, 188 and 150 for the BT549, MCF7, BT20 and UACC812 cell lines respectively. We obtained 191, 21, and 72 true positive BNs for BT549, MCF7, and BT20 cell lines respectively with an optimal fit to the data. For the UACC812 cell line, we were unable to obtain true positive BNs within the 7 day time limit for verification. Hence, we kept the first 20 BNs from the 150 ASP-optimal BNs for the UACC812 cell line. The *caspo-ts* method uses the ASP solver (clingo), which is able to exhaustively enumerate all solutions. The clingo solver by default uses an enumeration scheme, in which, once a solution is found, it backtracks to the first point from where the next solution can be found. This typically leads to the situation where successive solutions only change in a small part. As a result, *caspo-ts* may enter a solution space where BNs are clustered together. We have observed that given the size of the PKN and the small number of perturbations in the experimental data, the solution space of the *caspo-ts* can be rather very large containing billions of BNs making it difficult to enumerate true positive BNs (because of the model checking overhead) in a reasonable time if it gets stuck in a cluster of false positive BNs.

**Fig 3:**
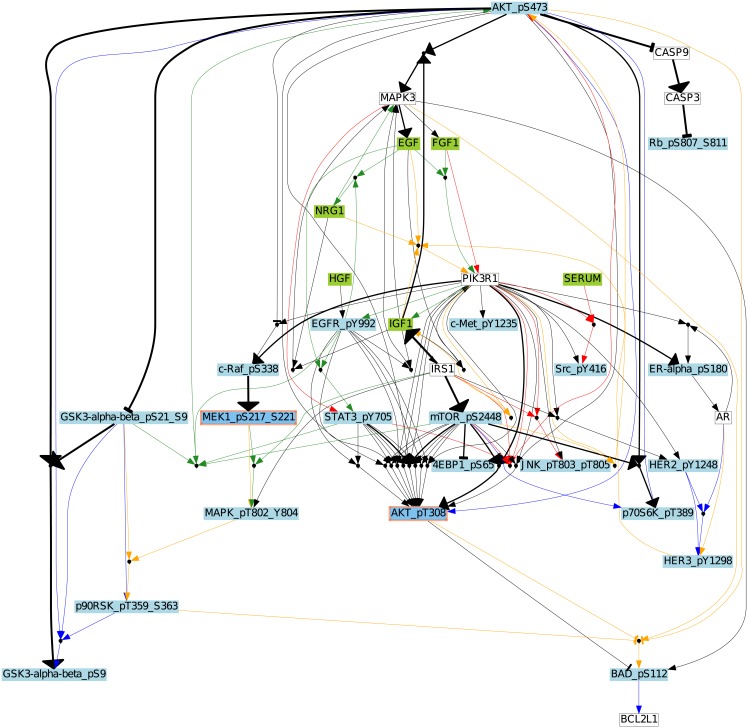
Boolean Network of breast cancer cell lines. The aggregated graph for all cell lines. Blue, red, green and orange edges are used for each cell line BT20, BT549, MCF7 and UACC812, respectively. The nodes are connected by logic gates (AND or OR) to their direct predecessors. Edges are used to show influences (→ for positive and ⊣ for negative). An AND gate is depicted by a small black circle where the incoming edges correspond to the inputs of the gate. An OR gate is depicted by multiple incoming edges to the node. A different color scheme is used to represent different types of nodes. The green color is for stimuli, the red for inhibitors, the blue for readouts, and the white for unobserved nodes. Black edges denote common hyper-edges across cell lines; the thickness of the black hyper-edge denotes the number of cell lines sharing this hyper-edge.

An aggregated network was built (Fig 3) by combining the BN families (with 191, 21, 72, and 20 BNs for BT549, MCF7, BT20, and UACC812 cell lines respectively) obtained for the four cell lines by keeping the hyper-edges (Boolean functions) having a frequency higher than 0.3 within each BN family. The frequency is calculated by counting the number of common Boolean functions and dividing it by the total number of Boolean functions within the BN family of each cell line. This aggregated network contains 34 nodes and 74 Boolean functions involving 36 AND gates. As compared to the PKN (Fig 2), the inferred networks are highly specific to each cell line. In Fig 3, all cell lines share only 4% of Boolean functions which are shown in thick black colored edges. This shows that the inferred BNs of these four breast cancer cell lines are very diverse and different from each other.

To measure cell lines similarity, we calculated the similarity score by applying the Graph Similarity Measure (see Materials and methods) on the family of BNs (with 191, 21, 72, and 20 BNs for BT549, MCF7, BT20, and UACC812 cell lines, respectively). This algorithm receives two parameters as input: (1) one gold standard BN and (2) a family of BNs. It outputs a score in [0; 1], measuring the average of the similarity scores between each BN in the family and the gold standard BN. In our case, the gold standard BN is the aggregation of one family of BNs. The similarity scores between all pairs of breast cancer cell lines are shown in Table 1. Fig 3 agrees with the results presented in Table 1 as we can see the clear discrepancies among the four cell lines. It can be seen that 23% of the Boolean functions are shared among BT549 and MCF7, and also between BT20 and UACC812. BT20 shares the least number of Boolean functions (15%) with BT549. This table revealed pronounced differences among different cell lines of breast cancer. We also analyzed the diversity of Boolean functions among the family of BNs within the same cell line. The similarity among Boolean functions from BT20 (0.73) and MCF7 (0.63) is higher than the ones from BT549 (0.43) and UACC812 (0.46) cell lines.

**Table 1:** Similarity scores among breast cancer cell lines.

Cell Lines	Size of BNs’ family	Similarity Score			
		BT20	BT549	MCF7	UACC812
*BT*20	72	0.73	0.15	0.17	0.23
*BT*549	191	**	0.43	0.23	0.20
*MCF*7	21	**	**	0.63	0.21
*UACC*812	20	**	**	**	0.46

### Heterogeneity among cell lines

**Fig 4:**
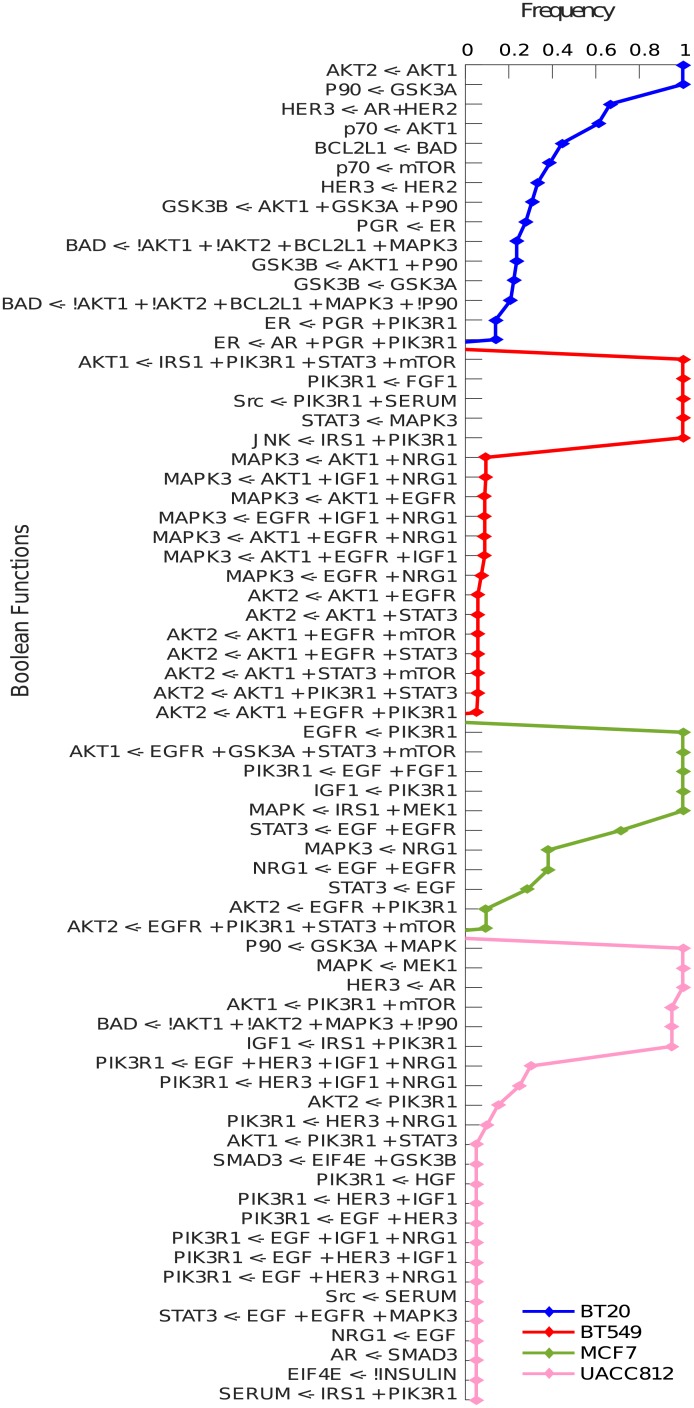
Heterogeneous Boolean functions. The Boolean functions are represented on the y-axis and the frequency of each Boolean function is shown on the x-axis. A Boolean function, or hyper-edge, is of the form *node* ← *expr*, where *node* is the receiver of the Boolean clause *expr* in the BN. In the Boolean clause, the *not* operator is represented by a “!” symbol and the AND operator by a “+” symbol. The disjunction of clauses is represented by multiple reactions upon the same receiver node.

There are a total of 69 distinct Boolean functions shown in Fig 4 along with their respective frequencies. It is interesting to note that the B549 and UACC812 cell lines have more distinct models among their family of BNs with a variable frequency range. This shows that these cell lines have different mechanisms agreeing with the results obtained through graph similarity measure given in Table 1.

**Fig 5:**
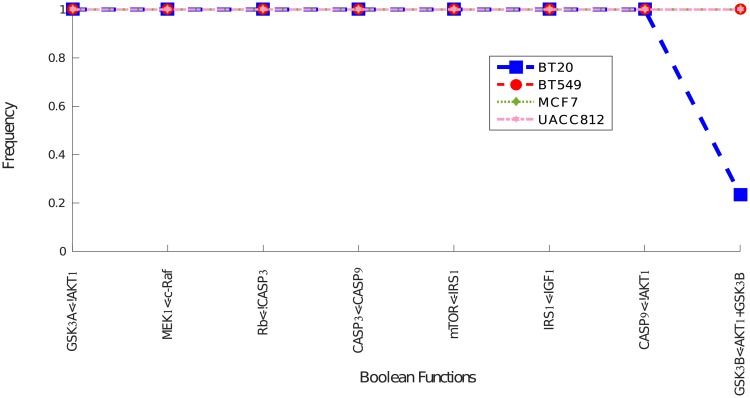
Common Boolean functions across all four cell lines. The Boolean functions are represented on the x-axis and the frequency of each Boolean function is shown on the y-axis.

Fig 5 shows the common Boolean functions along with their frequency in all BNs. Interestingly, only 4% of the Boolean functions are shared in all cell lines and 88% of these shared functions have the same frequency. In this figure, there is only one Boolean function which is frequent in 3 cell lines and has a lower frequency in BT20.

### Literature knowledge about Boolean functions discovered by *caspo-ts*

The union of the BNs learned for each cell line is displayed in the Supplementary Figures (S1, S2, S3 and S4 Figs). The *caspo-ts* method revealed that cell line specific reactions are clustered around the *AKT*, *MAPK3*, and *PIK3R1* proteins. *PI3K* is an important factor for cancer development in HER2 amplified cancers (UACC812) as compared to non-HER2 amplified (BT20, BT549 and MCF7) cancer cell lines. We can see from the Supplementary Figures (S1, S2, S3 and S4 Figs) that *PIK3R1* exists in all cell lines but is rather more connected in the UACC812 cell line with 10 incoming edges while in others with only 1 incoming edge. The *PIK3R1* node in UACC812 (S4 Fig) has a centrality measure of 0.37 while in the other three cell lines the centrality measure is less than 0.11. The centrality measure is used to quantify the most important node within a network i.e., the number of times a node has been used as a bridge (along the shortest path) to connect to other nodes in the network [27].

It has been established that *P1K3R1* (the regulatory unit of PI3K) plays an important role in suppressing tumors [28, 29]. Recently, it has been found that *PIK3R1* is mutated in 3% of breast cancer cell lines[30]. Nonetheless, it is worth studying the impact of the *PIK3R1* regulatory unit in breast cancer.

### Evaluation

The performance of the *caspo-ts* method is evaluated using three criteria: 1) RMSE calculation using a typical learning and testing data approach, 2) random data comparison, 3) AUROC (Area Under the Operating Curve) score.

The BNs are learned using the learning dataset (see Materials and methods) only. The prediction accuracy is evaluated by comparing the RMSE of trajectories in the testing dataset with those recovered by the learned networks (see Eq 1). There are two types of RMSE—discrete and model. The *discrete RMSE* is imposed by the discretization of the method. Since we use a discrete learning approach, our recovered traces will be in {0,1} and this introduces an error with respect to continuous measurements in [0;1]. The *model RMSE* refers to the learned BN error with respect to the normalized time series data; that is, the model RMSE is at least as large as the discrete RMSE. When the difference between these two is zero then the inferred BNs are able to recover the discrete trajectories without any error. If the model RMSE is greater than the discrete RMSE then the inferred BNs have some errors in the recoverability of the discrete time series data. To check how our method performs in case of random time series, we have calculated the *RMSE* score for random data and compared it with learning and testing data. Next, the validity of these networks is verified by comparing them with the canonical MTOR signaling pathway using two parameters, *i*.*e*., true positive rate (TPR) and false positive rate (FPR).

#### Validation using root mean square error criteria

The goal was not only to infer optimal BNs but also to verify that these BNs are able to recover trajectories that do not exist in the learning data. For this purpose, we use experimental testing data to check the specificity of the trajectories of the proposed networks. This testing data is provided by the HPN-DREAM challenge organizers (see Materials and methods). Table 2 shows the corresponding RMSE in case of learning and testing data. It can be seen that the inferred BNs are able to produce the trajectories without any error in the learning dataset for all cell lines. It is encouraging to see that the inferred BNs are able to recover the discrete testing trajectories without any error in MCF7, and with a minimal error of 0.0009, 0.0106, and 0.0094 in BT20, BT549, and UACC812, respectively.

**Table 2:** Root mean square error. This table summarizes the RMSE results for each cell line. We have calculated the discrete RMSE (error related to the discretization of the data) and the model RMSE (*caspo-ts* error). The Delta column shows the difference between model and discrete RMSE.

Cell Line	Learning			Testing		
	Discrete	Model	Delta	Discrete	Model	Delta
*BT*20	0.3464	0.3464	0	0.3293	0.3302	0.0009
*BT*549	0.3498	0.3498	0	0.3007	0.3113	0.0106
*MCF*7	0.3207	0.3207	0	0.2772	0.2772	0
*UACC*812	0.3464	0.3464	0	0.3084	0.3178	0.0094

We also compared the RMSE score with the top two best performers of the HPN-DREAM challenge. We got the top position with an RMSE score of 0.31 as compared to their RMSE scores of 0.47 and 0.50. Notice that in comparison to other HPN-DREAM challenge methods based on Bayesian inference, Regression, and Granger Causality among others, *caspo-ts* does not make new predictions but it checks the recoverability of the testing trajectories with the inferred BNs.

#### Validation using random data samples

**Fig 6:**
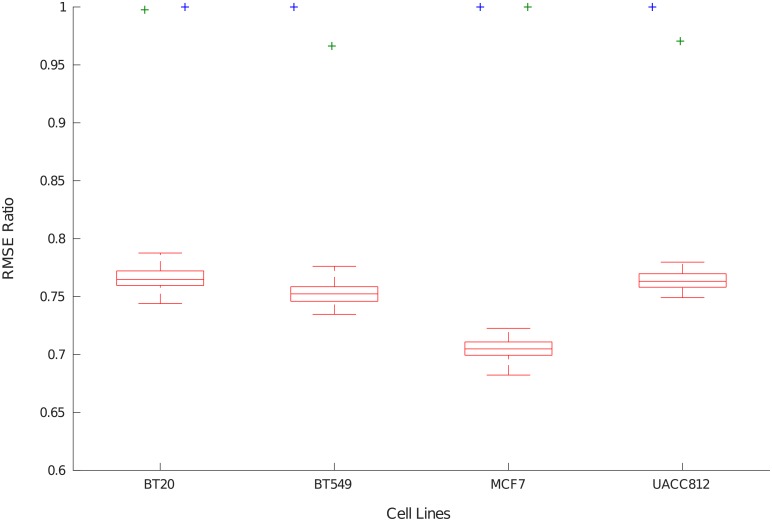
Performance assessment with learning, testing and random datasets. The x-axis shows the cell line and the y-axis shows the RMSE ratio (see Eq (2)) of the inferred BNs from the HPN-DREAM data for each cell line with respect to the three datasets. The three datasets are encoded by different color codes. The RMSE ratio with respect to the HPN-DREAM learning and testing datasets is shown in blue and green colors, respectively. The random dataset RMSE ratio distribution is shown as red boxplots.

The objective of this analysis is to show that the BNs obtained with *caspo-ts* using the HPN-DREAM datasets for the four cell lines have a worse RMSE score with respect to random trajectories, and therefore are very specific to the HPN-DREAM datasets. For this purpose, we generated 100 random data samples per cell line. In each sample, we generated a random value in [0; 1] for each readout protein in each time point without changing the perturbations. Then, we calculated the RMSE of these samples with respect to the inferred BNs of each cell line, and finally compared it with the learning and testing RMSE of these BNs. Fig 6 plots the RMSE ratio (see Eq (2)) of the inferred BNs with respect to the learning, testing and random data.

$$\begin{array}{c} RMSE\;ratio=\frac{Discrete\;RMSE}{Model\;RMSE} \end{array}$$ (2)

In Fig 6, the RMSE ratio for random datasets is displayed by red boxplots for each cell line, and the RMSE ratio for testing and learning datasets is shown as clear outliers in green and blue colors respectively. It is worth noting that the *caspo-ts* method has failed to recover random data time series, hence proving the specificity of the learned networks with respect to the HPN-DREAM challenge dataset.

Additionally, we computed the RMSE of the testing data by using a *leave one out* approach. For this we generated slightly modified samples, by selecting random values of 5% of the learning data points. The same experimental perturbations and readout proteins were kept. Our results show that the BNs learned from the 5% randomized data have an RMSE of 0.3113 with respect to the testing data, demonstrating *caspo-ts* robustness. For such 5% modified datasets, true positive BNs are difficult to obtain with the model checker; most candidates were false positive models. This highlights the complexity of this BN learning problem when few experimental perturbations are given because the space of candidate ASP-optimal BNs to verify is large and it is heavily populated with false positive Boolean models. Please refer to the supplementary information for details S2 Text.

#### Validation using MTOR canonical pathway

To perform the validation of the structure of the BNs, we calculated a set of *standard nodes* from our PKN which are downstream nodes of MTOR and belong to the canonical MTOR pathway. We then evaluated how many of these standard nodes are also downstream nodes of MTOR in the learned BNs. In the following, the set of downstream nodes of MTOR in the learned BNs is referred to as *inferred set*. The *inferred set* is specific to each cell line. True positive rate (TPR) and false positive rate (FPR) are defined by Eqs (3) and (4) respectively:

$$\begin{array}{c} TPR=\frac{TP}{TP+FN} \end{array}$$ (3)

$$\begin{array}{c} FPR=\frac{FP}{FP+TN} \end{array}$$ (4)

Here, TP is the number of nodes in the intersection between standard and inferred sets, FP is the number of nodes in the inferred set but not in the standard set, FN is the number of nodes in the standard set but not in the inferred set and TN is the number of nodes which are not in the standard set nor the inferred set. Note that TP and FP should not be confused with true and false positives from the over-approximation here.

**Fig 7:**
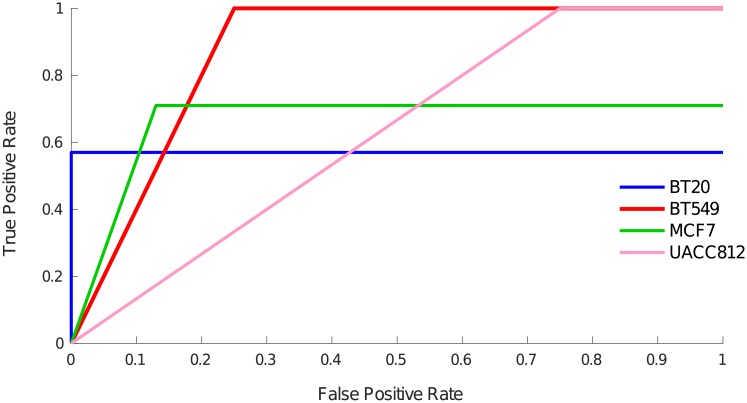
ROC curve across all cell lines. The x-axis shows the false positive rate and the y-axis denotes the true positive rate. These rates are calculated using Eqs (3) and (4). The average AUROC score is 0.77.

Fig 7 shows the Receiver Operating Characteristic (ROC) curve of each cell line. For BNs of each cell line, TPR and FPR was calculated using Eqs (3) and (4). BT549 cell line models are the most accurate ones followed by MCF7 and BT20. We can observe the clear distinction between true positive and false positive BNs. The BNs inferred by *caspo-ts* have an average AUROC score of 0.77 which is comparable to the AUROC score of 0.78 of the top performing method of HPN-DREAM challenge. A number of assumptions made during the modeling phase may have influenced our ranking. First, since our method can pinpoint the noisy, incomplete and erroneous experiment, it allows us to use only the reliable experimental settings. Second, our method constrains its solutions space to the proteins existing in the PKN, anything outside the prior knowledge cannot be found. From Fig 7, we can see that the *caspo-ts* method shows promising results for the inferred true positive BNs.

## Discussion

In this paper, we built cell line specific signaling networks for the DREAM time series dataset of 4 breast cancer cell lines (BT20, BT549, MCF7, and UACC812) using *caspo-ts*. This method combines Answer Set Programming and Model Checking techniques to infer true positive BNs verifying the experimental data. *Caspo-ts* allowed us to handle a midscale PKN (64 nodes and 178 edges) and a real dataset subject to experimental error. *Caspo-ts* enabled us to learn key dynamic mechanisms within the BNs explaining the time series data. Our results suggest that the behavior of cell line specific signaling networks is highly variable even under the same perturbations, agreeing with the heterogeneity of breast cancer and specifically with previous analysis on this data [21]. The inferred Boolean models of each cell line were analyzed to identify commonalities as well as discrepancies. Moreover, these inferred models can be executed computationally to identify potential drug targets or to see the effect of unseen perturbations. The predictive power of these models can be increased with improvements in protein interaction databases and comprehensive experimental data.

We have discovered 38% of the cell line dependent behaviors as compared to the 33% of the HPN-DREAM challenge winner [31]. We have implemented an algorithm to analyze the variability among cell lines and observed pairwise similarities among these cell lines. The similarity index varies from 15% (BT20 & BT549) to 23% (MCF7 & BT549, BT20 & MCF7). We have analyzed the similarity among the family of BNs of the same cell line as well, which varies from 43% to 73%. We have evaluated the accuracy of our method with RMSE and AUROC scores. The average RMSE of the inferred BNs was 0.31 placing *caspo-ts* in first place in comparison with the top scores reported in the HPN-DREAM challenge. Various choices made during this study may have an impact on the final score. The *caspo-ts* method allowed us to remove noisy and faulty experiments, leaving us with the reliable experimental settings only. Here, we made the choice to use only the reliable experiments of the learning dataset instead of using all experimental settings. Also, we did not observe all 45 proteins as we could not find connections in our PKN for all the studied proteins, leaving us with approximately 23 proteins for each cell line.

Nonetheless, the obtained results are quite promising, making *caspo-ts* a good candidate computational method for learning models given time series datasets and a prior knowledge network. In addition, *caspo-ts* can be used to pinpoint the errors in the experimental data. In particular, we discovered four experiments where the protein *AKT* was inhibited and had a dynamic behavior as a readout protein. Our work therefore provides a novel approach to show erroneous experiments which is crucial and complementary to current approaches. Finally, the HPN-DREAM dataset contained some noisy readings of experiments. Noisy experimental data reduces the efficiency of computational methods by increasing the variability among constructed Boolean models. To overcome this, we suggest to build automated methods to filter out the noisy experiments. This approach provides a step forward in building context dependent networks in the case of phosphoproteomic data.

### Perspective

As a future direction, we are planning to investigate several aspects of the *caspo-ts* method, such as (i) the order of the solution space of over-approximated Boolean models; (ii) the computational time for checking reachability; (iii) designing an efficient experimental design strategy and applying it prior to selecting the most informative experiments. Because *caspo-ts* uses an ASP solver to enumerate BNs, in the resulting sequence of solutions similar BNs are typically clustered together. This can be problematic for large scale problems where we cannot explore the whole solution space in reasonable time. We are currently working on sampling to randomly select BNs from the solution space. Further, we are also studying another technique, which allows for shuffling the order in which solutions are enumerated [32]. We are planning to implement this by dynamically modifying the heuristic of the ASP solver at execution time. Finally, to reduce the false positive rate, we are planning to use multi-shot ASP solving [33] allowing us to customize the search and modify the underlying ASP program at runtime. In our case, we can call the model checker during solving to learn and add constraints to prune wrong BNs early on.

## Materials and methods

### Data acquisition

The DREAM portal provides unrestricted access to complex, pre-tested data to encourage the development of computational methods. In this study, we are focused on the HPN-DREAM challenge, which was motivated by the fact that the same perturbation may lead to different signaling behaviors in different backgrounds, making it necessary to build a model which can perform unseen predictions (absent from the learning data). The main goal of the HPN-DREAM challenge is to learn signaling networks efficiently and effectively to predict the dynamics of breast cancer [19].

#### Learning data

Reverse Phase Protein Array (RPPA) quantitative proteomics technology was used for generating the dataset of this challenge. The measurements focus on short term changes on up to 45 proteins and their phosphorylation over 0 to 4 hours. The HPN-DREAM dataset includes temporal changes in phosphorylated proteins at seven different time points (*t*_1_ = 0min, *t*_2_ = 5min, *t*_3_ = 15min, *t*_4_ = 30min, *t*_5_ = 60min, *t*_6_ = 120min, *t*_7_ = 240 min). The learning data consists of four cancer cell lines (BT20, BT549, MCF7 and UACC812) under different perturbations (≈8 stimuli and ≈3 inhibitors). The number of perturbations varies from 24 to 32 depending on the cell line. In each cancer cell line approximately 45 phosphorylated proteins are measured against different sets of perturbations over multiple time scales. After removing perturbations with inconsistent behaviors or incomplete time series, we had 15, 13, 13 and 18 perturbations for MCF7, BT20, BT549 and UACC812 cell lines respectively measuring 23 readouts.

#### Testing data

Test data is available for assessing the performance of networks learned from the learning data. The HPN-DREAM portal provides testing data for four cancer cell lines (BT20, BT549, MCF7 and UACC812) under different perturbations (8 stimuli and 1 inhibitor). They contain gold standard datasets of time series predictions of up to 45 proteins having the same time scale as learning data [19–21]. The number of perturbations varies from 7 to 8 depending on the cell line. This data is used to test the quality of the BNs given by *caspo-ts*.

#### Normalization

The protein measurements were ranging over variable ranges. Maximum value based normalization was used to set the measurements between a common scale, *i*.*e*., 0 and 1 in order to assign activation or inactivation values to variables or species of the BN. Eq (5) describes the formula used for the normalization. Given time series *T*^*P*^, we obtain time series *T*′^*P*^:

$$\begin{array}{c} {\left({t^{\prime }}_{j}^{P}\right)}_{i}=\frac{{\left(t_{j}^{P}\right)}_{i}}{\max\{{\left(t_{l}^{Q}\right)}_{i}|Q\in P,1\leq l\leq k\}} \end{array}$$ (5)

where *i* ∈ {1, …, *m*} are the observations, *j* ∈ {1, …, *k*} are time-points, and P∈P are the perturbations. Here ${\left(t_{j}^{P}\right)}_{i}$ represents the value of protein *i* under perturbation *P* at time-point *j* and the denominator denotes the highest value of protein *i* under all perturbations and time-points.

### Prior knowledge network derivation

PKNs are available in different databases such as Reactome, PID, and KEGG among others [26, 34–45]. We can construct a PKN through a tool such as ReactomeFIViz [25] which is available as a Cytoscape [46] plugin. A PKN alone cannot be used to build reliable dynamical models or to explain underlying biological behaviors [16, 47], especially in the case of multiple perturbations data because of the need of specificity. In order to overcome this issue, methods have been proposed which take into account both literature based knowledge and experimental data to build logic models [3, 7, 11, 12, 16].

### Learning Boolean Networks with *caspo-ts*

In the *caspo-ts* modeling framework section, we have given the formal definitions of the inputs and the output (BN) of the *caspo-ts*. Here, we formally describe the over-approximation criteria. Finally. we give pseudo encodings of the input of the ASP part of the *caspo-ts*.

#### Over-approximation criteria

The goal is to generate BNs that can reproduce the experimental data as well as possible. For this objective, the states have to be reachable from another. We use *x* →* *y* to say that state *y* can be reached from state *x* with an arbitrary number of steps. Since this reachability is a computationally hard problem (PSPACE-complete) [48], we use an over-approximation for checking reachability resulting in false positive (FP) BNs [7, 8]. The meta-states have been introduced to check over-approximated reachability.

A meta-state *u* = (*u*_1_, *u*_2_, …, *u*_*n*_) is a vector of dimension *n* over non-empty subsets of B, noted M={{0},{1},{0,1}}; the set of meta-states is $M^{n}$. Meta-states characterize a set of Boolean states: a state $x\in B^{n}$ belongs to a meta-state *u*, written *x* ∈ *u*, iff each Boolean component *x*_*i*_ belongs to the set *u*_*i*_. Given a state *x*, we use $\bar{x}$ for the corresponding meta-state ({*x*_1_}, …, {*x*_*n*_}). We define the transition relation *u* ⇉ *v* between the meta-states *u* and *v* as follows: *u* ≠ *v* and *v* = (*u*_1_, …, *u*_*i*_ ∪ {*f*_*i*_(*x*) ∣ *x* ∈ *u*}, …, *u*_*n*_) for some 1 ≤ *i* ≤ *n*.

In [8], it has been shown that if *y* is reachable from *x* (*x* →* *y*) then there exists a meta-state *u* such that *y* ∈ *u* and $\bar{x}\;{⇉}^{*}\;u$. This definition is further refined to describe the necessary condition for reachability called support consistency. A state *x* is support consistent with state *y* denoted by *x* ⇝* *y*, if and only if there exists a meta-state *u* with $\bar{x}\;{⇉}^{*}\;u$ such that *y* ∈ *u* and for all 1 ≤ *i* ≤ *n* either

- *y*_*i*_ ≠ *x*_*i*_, or
- *y*_*i*_ = *x*_*i*_ and *u*_*i*_ ≠ {0, 1}, or
- *y*_*i*_ = *x*_*i*_, *u*_*i*_ = {0, 1}, and there exists *z* ∈ *u* such that *f*_*i*_(*z*) = *y*_*i*_.

If state *y* is reachable from state *x* (*x* →* *y*) then *x* ⇝* *y*. Since we are using the over-approximation criteria, it is possible that some BNs may fail to reproduce the exact trajectories of the time series data. These BNs are called false positive (FP). To filter out the false positive BNs, exact model checking is applied.

#### Input encodings

Here, we provide the pseudo logic program to describe the input data given in the *caspo-ts* modeling framework Section. The logic program in written in the ASP language. ASP is a powerful declarative logic programming language for knowledge representation and reasoning [49]. The basic idea is to encode the problem using a non-monotonic logic program and then feed it into the ASP solver, which computes the solution of the problem in the form of models (also known as answer sets). Note that we provide encodings only for the input data here, please refer to supplementary information for details (S1 Text).

Facts, rules and constraints are the building blocks of ASP programs. Here we use facts to describe the inputs. The PKN (*V*, *E*, *σ*) is described by the following facts:

$$\begin{array}{ll} node\left(v\right). & \text{for}\;v\in V\\ edge\left(u,v,s\right). & \text{for}\;\left(u,v\right)\in E\;\text{and}\;\left(\left(u,v\right),s\right)\in \sigma \end{array}$$

For each perturbation P∈P and phosphoproteomic time series *T*^*P*^, we have the following facts:

$$\begin{array}{ll} clamped\left(P,v,0\right). & \text{for}\;v\in P\cap I\\ clamped\left(P,v,1\right). & \text{for}\;v\in P\cap S\\ obs\left(P,j,v_{i},s\right). & \text{for}\;s={\left(t_{j}^{P}\right)}_{i},1\leq j\leq k\;\text{and}\;1\leq i\leq m \end{array}$$

#### Available software

The *caspo-ts* github repository contains the sources as well as detailed user guide with two examples at the following address: https://github.com/misbahch6/caspo-ts.

### Graph similarity measure

This work introduces the study of a graph similarity measure in order to check the variability among the families of BNs generated by *caspo-ts*. We compare the reactions existing in the gold standard network (*A*) with the family of BNs (B) and is based on the Jaccard similarity coefficient which measures the similarity of these models.

#### Jaccard similarity coefficient

The Jaccard index between *A* and *B*_*i*_ can be defined as length of the intersection divided by the union:

$$\begin{array}{c} J\left(A,B_{i}\right)=\frac{|A\cap B_{i}|}{|A\cup B_{i}|}=\frac{|A\cap B_{i}|}{|A|+|B_{i}|-|A\cap B_{i}|} \end{array}$$ (6)

We apply the Jaccard Similarity Coefficient on *B*_*i*_ (where $B_{i}\subset B$) by taking A as being the gold standard.

## Supporting information

S1 Fig Union of BNs of BT20. Here, we show the union of BNs for the cell line BT20. This network is generated by combining 72 true positive BNs. It contains 31 nodes and 41 boolean functions with 12 AND gates. There are 2 stimuli, 2 inhibitors and 21 readouts.
(PDF)

S2 Fig Union of BNs of BT549. Here, we show the union of BNs for the cell line BT549. This network is generated by combining 191 true positive BNs. It contains 28 nodes and 53 boolean functions with 35 AND gates. There are 5 stimuli, 2 inhibitors and 17 readouts.
(PDF)

S3 Fig Union of BNs of MCF7. Here, we show the union of BNs for the cell line MCF7. This network is generated by combining 21 true positive BNs. It contains 24 nodes and 37 boolean functions with 19 AND gates. There are 4 stimuli, 2 inhibitors and 15 readouts.
(PDF)

S4 Fig Union of BNs of UACC812. Here, we show the union of BNs for the cell line UACC812. This network is generated by combining 20 BNs. It contains 33 nodes and 54 boolean functions with 29 AND gates. There are 6 stimuli, 2 inhibitors and 18 readouts.
(PDF)

S1 Table Computation summary. Here, we show the number of verified solutions, true positive and false positive BNs, and their computation (ASP solving and Model Checking steps) time for each cell line. It is worth noting that we generated 32 true positive BNs for UACC812 cell line by allowing the model checker to run without bounding it to the 7 day time limit. The ASP solving was performed on a standard laptop machine. The model checking task was performed on a cluster with 560 cores and 1.9 Tb of RAM.
(PDF)

S1 Text ASP encodings. (PDF)

S2 Text Validation by introducing noise in the learning data. (PDF)
