# Supplementary figures and images for: Computational discovery of dynamic cell line specific Boolean networks from multiplex time-course data
Source: PLoS Comput Biol. 2018 Oct 29;14(10):e1006538. doi: 10.1371/journal.pcbi.1006538 (PMC6224120; doi:10.1371/journal.pcbi.1006538)

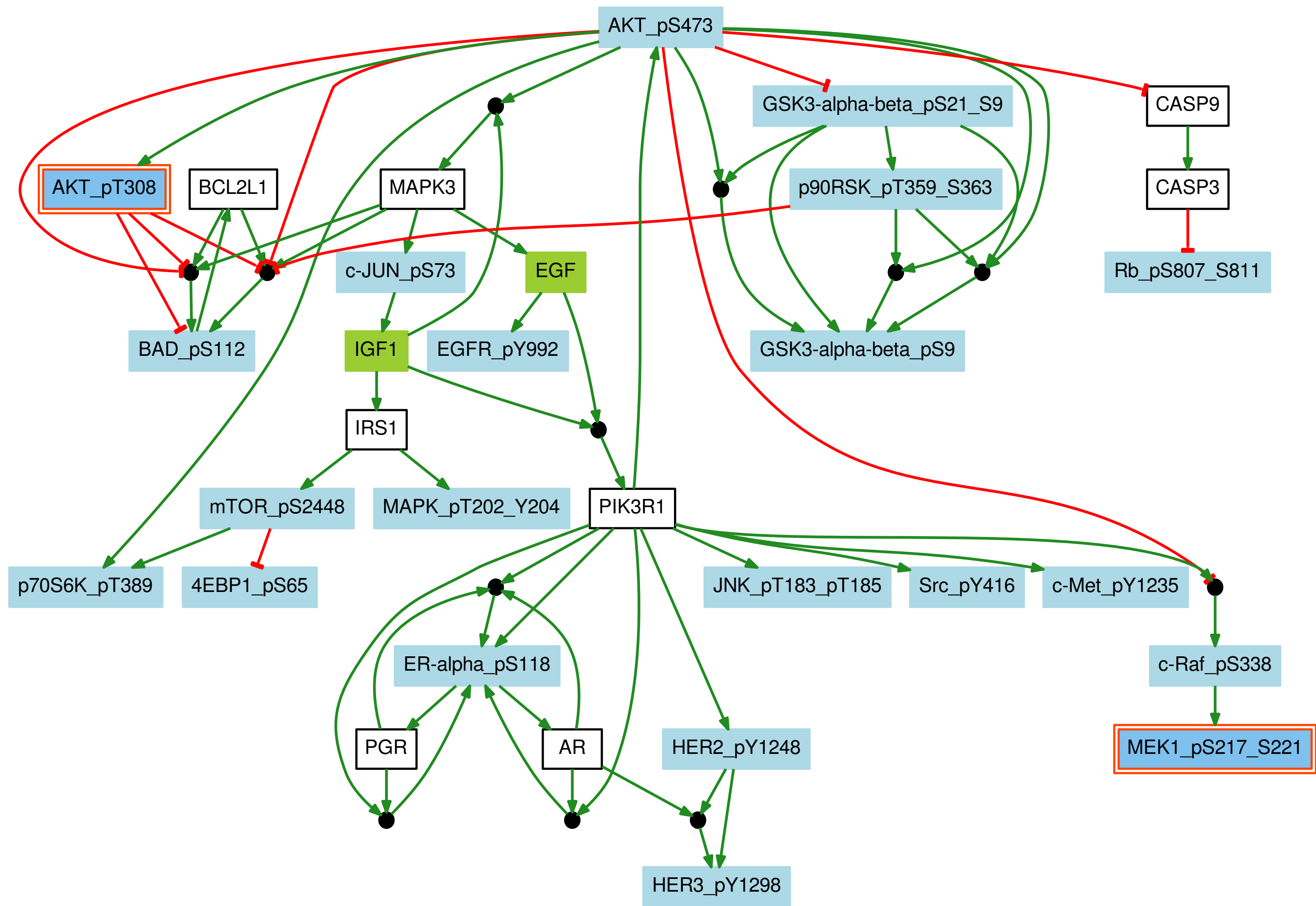

Supplement: S1 Fig — Here, we show the union of BNs for the cell line BT20. This network is generated by combining 72 true positive BNs. It contains 31 nodes and 41 boolean functions with 12 AND gates. There are 2 stimuli, 2 inhibitors and 21 readouts. (PDF) [file pcbi.1006538.s001.pdf]

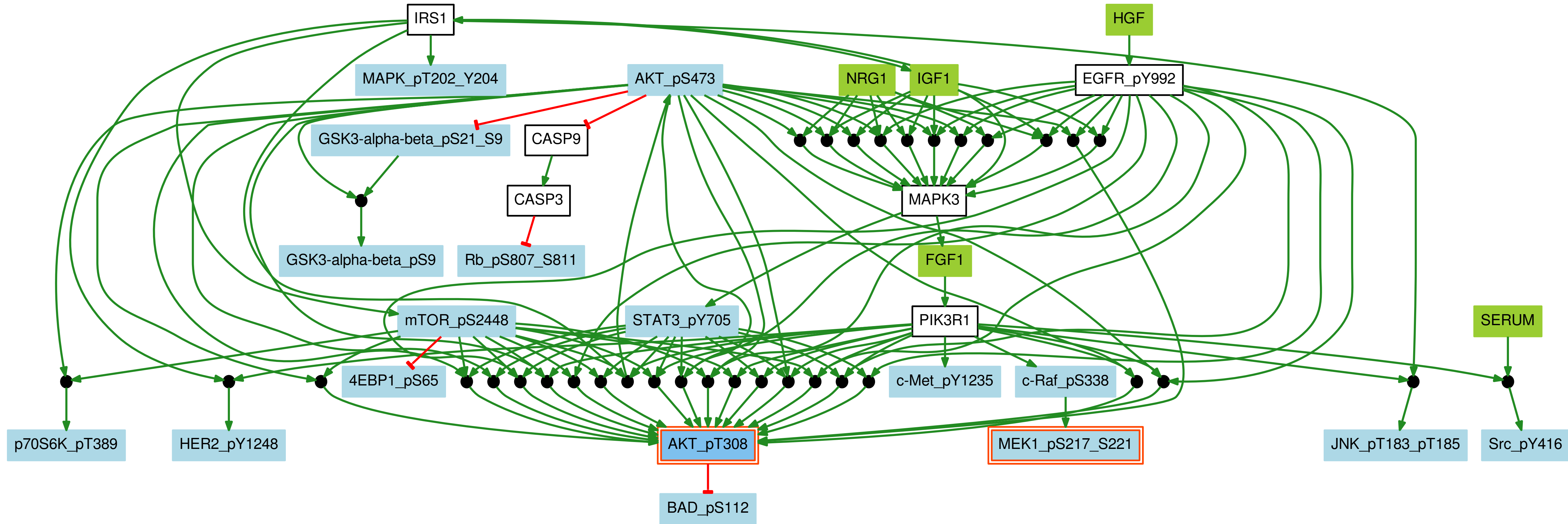

Supplement: S2 Fig — Here, we show the union of BNs for the cell line BT549. This network is generated by combining 191 true positive BNs. It contains 28 nodes and 53 boolean functions with 35 AND gates. There are 5 stimuli, 2 inhibitors and 17 readouts. (PDF) [file pcbi.1006538.s002.pdf]

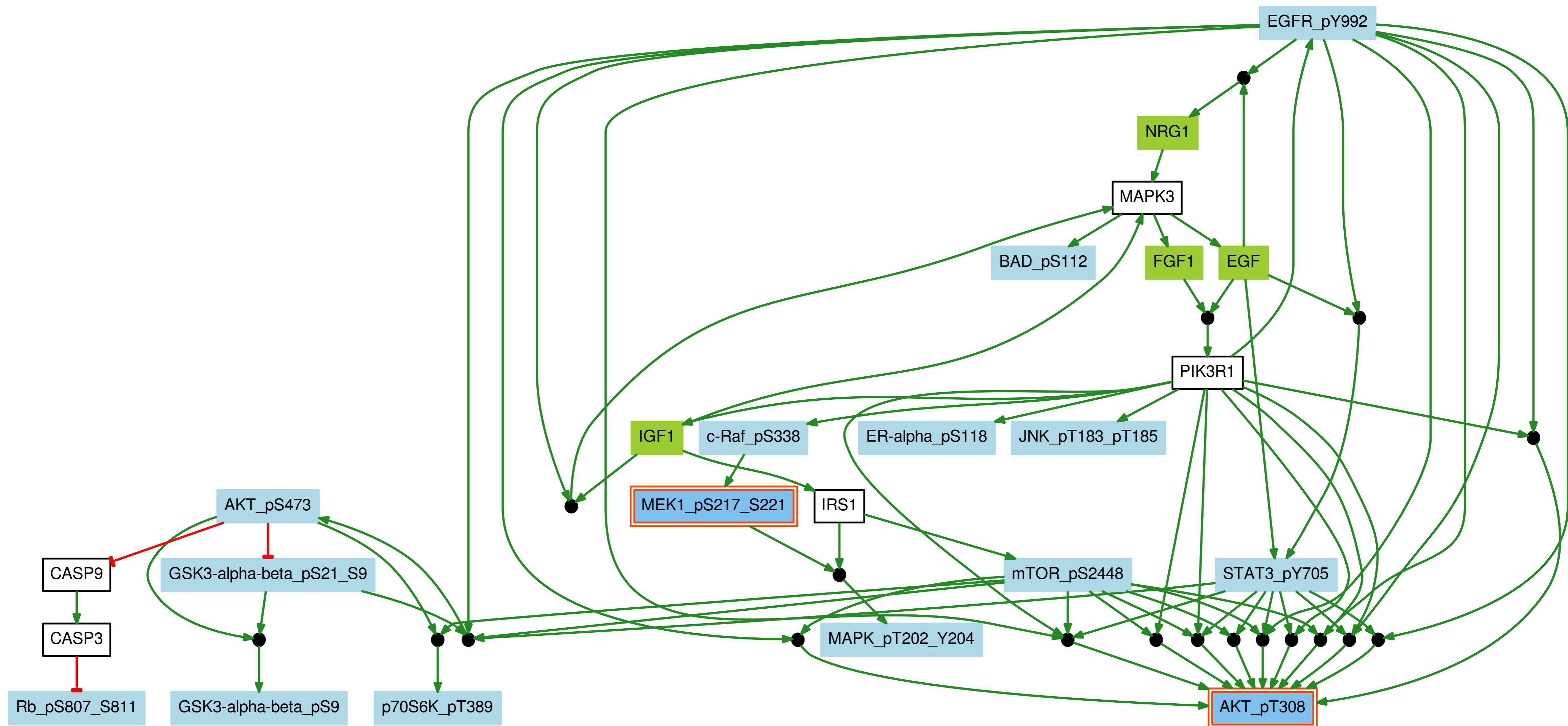

Supplement: S3 Fig — Here, we show the union of BNs for the cell line MCF7. This network is generated by combining 21 true positive BNs. It contains 24 nodes and 37 boolean functions with 19 AND gates. There are 4 stimuli, 2 inhibitors and 15 readouts. (PDF) [file pcbi.1006538.s003.pdf]

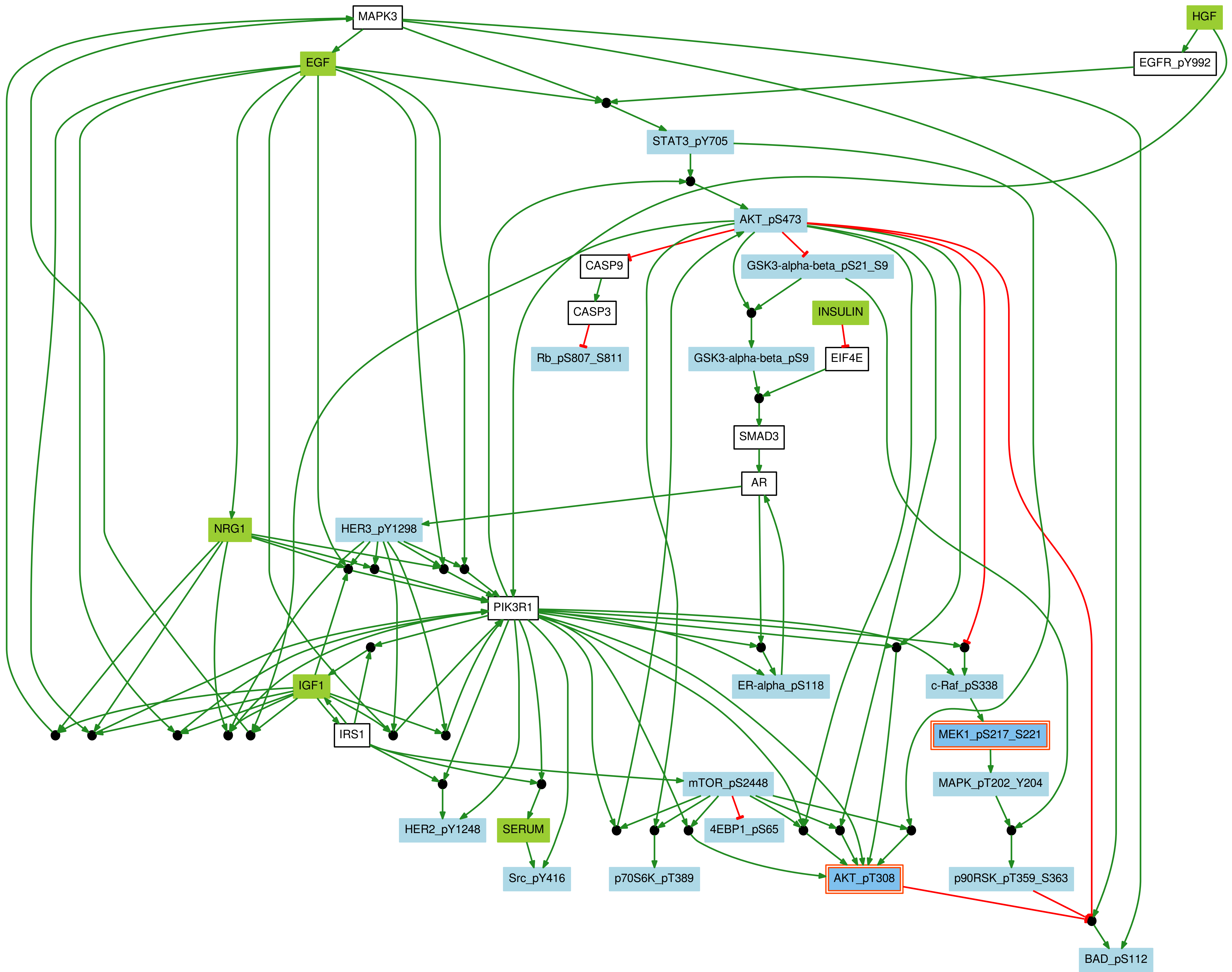

Supplement: S4 Fig — Here, we show the union of BNs for the cell line UACC812. This network is generated by combining 20 BNs. It contains 33 nodes and 54 boolean functions with 29 AND gates. There are 6 stimuli, 2 inhibitors and 18 readouts. (PDF) [file pcbi.1006538.s004.pdf]
