# Supplementary material for: Computational discovery of dynamic cell line specific Boolean networks from multiplex time-course data
Source: PLoS Comput Biol. 2018 Oct 29;14(10):e1006538. doi: 10.1371/journal.pcbi.1006538 (PMC6224120; doi:10.1371/journal.pcbi.1006538)
Supplement: S1 Table — Here, we show the number of verified solutions, true positive and false positive BNs, and their computation (ASP solving and Model Checking steps) time for each cell line. It is worth noting that we generated 32 true positive BNs for UACC812 cell line by allowing the model checker to run without bounding it to the 7 day time limit. The ASP solving was performed on a standard laptop machine. The model checking task was performed on a cluster with 560 cores and 1.9 Tb of RAM. (PDF) [file pcbi.1006538.s005.pdf]

Computation Summary

Cell Line	Number of Solutions	True Positives	False Positives	Time	
				ASP solving	Model Checker
$BT20$	188	72	116	210 seconds	7 days
$BT549$	231	191	40	93 seconds	7 days
$MCF7$	52	21	21	36 seconds	7 days
$UACC812$	150	0	150	197 seconds	7 days

S1 Table. Computation summary. Here, we show the number of verified solutions, true positive and false positive BNs, and their computation (ASP solving and Model Checking steps) time for each cell line. It is worth noting that we generated 32 true positive BNs for UACC812 cell line by allowing the model checker to run without bounding it to the 7 day time limit. The ASP solving was performed on a standard laptop machine. The model checking task was performed on a cluster with 560 cores and 1.9 Tb of RAM.
