# Supplementary material for: Computational discovery of dynamic cell line specific Boolean networks from multiplex time-course data
Source: PLoS Comput Biol. 2018 Oct 29;14(10):e1006538. doi: 10.1371/journal.pcbi.1006538 (PMC6224120; doi:10.1371/journal.pcbi.1006538)
Supplement: S1 Text — (PDF) [file pcbi.1006538.s006.pdf]

ASP Encodings

Here, we provide the pseudo encoding to describe the ASP solving problem given in the *caspo-ts* modeling framework section. After describing inputs in the *caspo-ts* modeling framework section and output in the materials and method section, we show how we filtered these models using the over-approximation criteria, we show the the objective function to optimize BNs. Please refer to [1, 2] for details.

Over-approximation

Here, we show how we filtered the compatible BNs with the over-approximation criteria. We start with generating Boolean values for each node of the BN with the following rule:

$$1\{state(P,T,U,(0;1))\}1 \leftarrow P \in \mathcal{P}, node(U), T = 1..k.$$

Here, state predicate is implemented as a choice rule. It has four arguments. The first argument represents the perturbation P, the second represents the time-point T, the third is the node U, and the fourth represents the guessed value for the the node U. States are subject to further constraints which are omitted here for brevity.

Further, we calculate the meta-states from the given states of the BNs with the following rules:

$$meta(P,T,U,V) \leftarrow state(P,T,U,V).$$
$$update(P,T,U,V) \leftarrow \text{“node U takes value V if its formula can evaluate to V”}.$$
$$meta(P,T,U,V) \leftarrow update(P,T,U,V).$$

The first rule initializes the meta-states from the *state* predicate. The second rule selects possible updates by evaluating the Boolean functions associated with the nodes. The third rule updates the current meta-state with the *update* predicate. We omit the exact encodings here and refer to the literature [1] for details.

The following integrity constraints encode the support consistency condition described in the Formal Description section:

$$\leftarrow state(P,T+1,U,V), \mathbf{not}\ meta(P,T,S,V).$$
$$\leftarrow state(P,T+1,U,V), \mathbf{not}\ update(P,T,U,V), update(P,T,U,W), V \neq W.$$

The first rule ensures that the state at time-point $T+1$ is in the meta-state reachable from time-point T. The second rule implements the condition in the third item in the definition of support consistency described in the Formal Description section. It states that if a state at time-point $T+1$ has value V and in the update predicate it has value W then there must exist an update in-between to recover the final value V.

Optimization

Here, we describe the objective function to minimize the difference between the phosphoproteomic time series data and the guessed time series for the BN. The following rules declared the objective function by using *#minimize* directive:

$$\#minimize\{M-49, P, T, U : obs(P,T,U,M), state(P,T,U,0), M \geq 50\}.$$
$$\#minimize\{50-M, P, T, U : obs(P,T,U,M), state(P,T,U,1), M < 50\}.$$

If the conditions after the : do not evaluate to true, then the guessed time series does not match with the corresponding measurements (phosphoproteomic data) and a penalty is accumulated by the minimize statement. Note that since ASP does not support continuous values, we converted them to integers in the range from 0 to 100 by using the formula: $\lfloor x * 100 \rfloor$.

References

1. Ostrowski M, Paulevé L, Schaub T, Siegel A, Guziolowski C. Boolean network identification from perturbation time series data combining dynamics abstraction and logic programming. Biosystems. 2016;149:139–153.

2. Videla S, Guziolowski C, Eduati F, Thiele S, Grabe N, Saez-Rodriguez J, et al. Revisiting the training of logic models of protein signaling networks with ASP. In: Computational Methods in Systems Biology. Springer; 2012. p. 342–361.
