# Supplementary material for: Computational discovery of dynamic cell line specific Boolean networks from multiplex time-course data
Source: PLoS Comput Biol. 2018 Oct 29;14(10):e1006538. doi: 10.1371/journal.pcbi.1006538 (PMC6224120; doi:10.1371/journal.pcbi.1006538)
Supplement: S2 Text — (PDF) [file pcbi.1006538.s007.pdf]

Validation by introducing noise in the learning data

The following analysis was performed for cell line BT549. Recall that checking the excat reachability of the BNs with respect to a binarized time series trace is a computationally expensive task. The time to model check BNs differs from one network to another. For this case study, the model checking time varied from minutes to days. Because of this reason, we have done this analysis for only one cell line.

We generated 18 modified learning datasets by randomly introducing 5% of noisy data in each dataset; that is, the 5% of the total time point values were generated randomly. We used these datasets to learn 231 ASP-optimal BNs. Then we passed these BNs to the model checker to detect true positive BNs. The model checker verified the exact reachability of 4158 BNs contained in 18 modified datasets. It detected 582 true positive BNs. To verify if these TP BNs can reproduce the time series traces existing in the testing dataset of HPN-DREAM challenge. We used the Equation (1) of the main paper to calculate the RMSE score. Our results (Table 1) show that we were able to obtain an RMSE of 0.3113 for BNs learned from 5% modified learning datasets. It shows as well, that most of the ASP-optimal BNs were false positive BNs. Interestingly, the Delta RMSE, which measures how well *caspo-ts* can recover the discrete time-series data, points to regions where the randomization of the learning data was important and therefore the ASP-optimal BNs did not reocover the full discretized time series data. Overall these results demonstrate that our learning process is robust and is able to handle noisy time series data.

Table 1. Computation Summary. This table summarizes the analysis performed on the BT549 cell line. The # of solutions column shows the number of ASP optimal BNs. The true and false positive columns show the number of true positive and false positive BNs returned by the model checker. The delta RMSE column shows the difference between the discrete and model RMSE. The ASP solving column represents the time spent on learning ASP optimal BNs for each sample. The model checking column shows the time spent on verifying the BNs for each sample. The last column RMSE represents the corresponding RMSE of each sample with respect to the testing data. The ASP solving was performed on a standard laptop machine. The model checking task was performed on a server with 1.5 Tb of RAM.

**BT549**	**# of Solutions**	**Learning Data**			**Time**		**Testing Data**
		True Positives	False Positives	Delta RMSE	ASP solving	Model Checking	RMSE
1	231	231	0	0	124 seconds	18 days	0.3113
2	231	231	0	0.0009	125 seconds	15 days	0.3113
3	231	0	231	0.0002	122 seconds	3 days	0.3113
4	231	0	231	0.0004	112 seconds	10 days	0.3113
5	231	0	231	0.0007	101 seconds	7 days	0.3113
6	231	0	231	0	110 seconds	9 days	0.3113
7	231	0	231	0	133 seconds	5 days	0.3113
8	231	0	231	0	133 seconds	6 days	0.3113
9	231	0	231	0.0003	134 seconds	6 days	0.3113
10	231	0	231	0	136 seconds	18 days	0.3113
11	231	0	231	0.0001	90 seconds	22 days	0.3113
12	231	0	231	0.0029	148 seconds	30 days	0.3113
13	231	0	231	0	104 seconds	30 days	0.3113
14	231	0	231	0.0003	91 seconds	23 days	0.3115
15	231	0	231	0.0001	329 seconds	22 days	0.3113
16	231	120	111	0.0018	100 seconds	25 days	0.3113
17	231	0	231	0	150 seconds	30 days	0.3113
18	231	0	231	0.0004	131 seconds	30 days	0.3115
